# 2-[(2-Aza­niumyleth­yl)carbamo­yl]phenolate–phenol (1/1)

**DOI:** 10.1107/S1600536813005849

**Published:** 2013-03-06

**Authors:** Sihem Yebedri, Samira Louhibi, Sofiane Bouacida, Ali Ourari, Thierry Roisnel

**Affiliations:** a Laboratoire de Chimie Inorganiue et d’Environment, Université def Tlemcen, BP 119, Tlemcen 13 000, Algeria; b Unité de Recherche de Cimie de l’Environnement et Moléculaire Structurale, CHEMS, Université Mentouri-Constantine, 25000 , Algeria; c Laboratoire d’Electrochimie, d’Ingénierie Moléculaire et de Catalyse Redox (LEIMCR), Faculté des Sciences de l’Ingénieur, Université Farhat Abbas, Sétif 19000, Algeria; d Centre de Difractométrie X, UMR 6226 CNRS Unité Sciences Chimiques de Rennes, Université de Rennes I, 263 Avenue du Général Leclerc, 35042 Rennes, France

## Abstract

In the title 1:1 adduct, C_9_H_12_N_2_O_2_·C_6_H_6_O, the dihedral angle between the benzene ring and the salicylic amide group is 6.68 (6)°. The conformation of the amide group is supported by two intra­molecular N—H⋯O hydrogen bonds, which close *S*(6) and *S*(7) rings. In the crystal, the components are linked by O—H⋯O and N—H⋯O hydrogen bonds, generating (100) sheets.

## Related literature

For background to salicylic amides as ligands, see: Koch (2001 ▶); Hancock & Martell (1989 ▶).
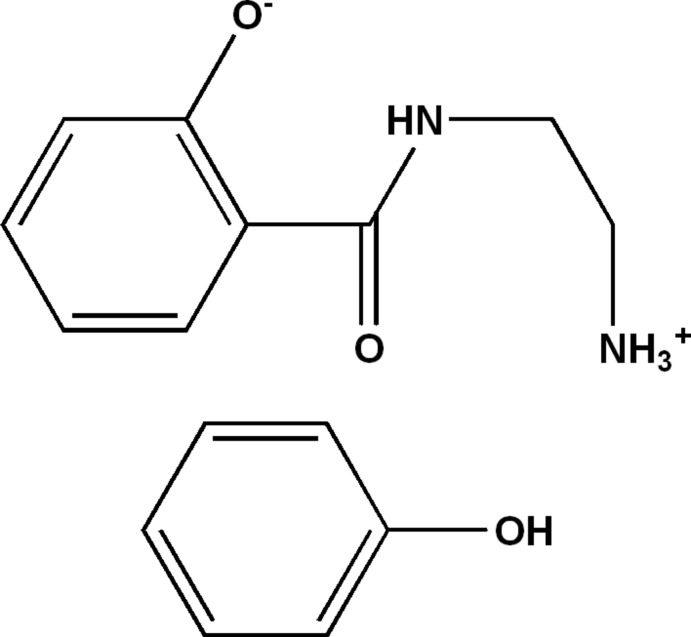


## Experimental

### 

#### Crystal data

C_9_H_12_N_2_O_2_·C_6_H_6_O
*M* *_r_* = 274.31
Monoclinic, 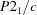

*a* = 12.6494 (4) Å
*b* = 13.2145 (6) Å
*c* = 8.5445 (4) Å
β = 100.637 (2)°
*V* = 1403.72 (10) Å^3^
*Z* = 4
Mo *K*α radiation
μ = 0.09 mm^−1^
*T* = 150 K
0.58 × 0.52 × 0.38 mm

#### Data collection

Bruker APEXII CCD diffractometer
Absorption correction: multi-scan (*SADABS*; Bruker, 2011 ▶) *T*_min_ = 0.860, *T*_max_ = 0.966
12244 measured reflections
3208 independent reflections
2649 reflections with *I* > 2σ(*I*)
*R*_int_ = 0.035

#### Refinement

*R*[*F* ^2^ > 2σ(*F* ^2^)] = 0.040
*wR*(*F* ^2^) = 0.102
*S* = 1.03
3208 reflections
181 parameters
H-atom parameters constrained
Δρ_max_ = 0.27 e Å^−3^
Δρ_min_ = −0.21 e Å^−3^

### 

Data collection: *APEX2* (Bruker, 2011 ▶); cell refinement: *SAINT* (Bruker, 2011 ▶); data reduction: *SAINT*; program(s) used to solve structure: *SIR2002* (Burla *et al.*, 2005 ▶); program(s) used to refine structure: *SHELXL97* (Sheldrick, 2008 ▶); molecular graphics: *ORTEP-3 for Windows* (Farrugia, 2012 ▶) and *DIAMOND* (Brandenburg & Berndt, 2001 ▶); software used to prepare material for publication: *SHELXL97*.

## Supplementary Material

Crystal structure: contains datablock(s) global, I. DOI: 10.1107/S1600536813005849/hb7050sup1.cif

Structure factors: contains datablock(s) I. DOI: 10.1107/S1600536813005849/hb7050Isup2.hkl

Supplementary material file. DOI: 10.1107/S1600536813005849/hb7050Isup3.cml

Additional supplementary materials:  crystallographic information; 3D view; checkCIF report

## Figures and Tables

**Table 1:** Hydrogen-bond geometry (Å, °)

*D*—H⋯*A*	*D*—H	H⋯*A*	*D*⋯*A*	*D*—H⋯*A*
O1—H1⋯O3^i^	0.82	1.87	2.6696 (13)	166
N1—H1*N*⋯O2	0.86	1.93	2.6490 (13)	140
N2—H2*A*⋯O1	0.89	2.21	2.8995 (14)	134
N2—H2*A*⋯O3	0.89	2.56	3.0547 (13)	116
N2—H2*B*⋯O2^ii^	0.89	1.93	2.7506 (13)	152
N2—H2*C*⋯O2^iii^	0.89	1.81	2.6939 (13)	174

Symmetry codes: (i) 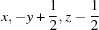; (ii) 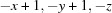; (iii) 
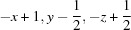
.

**Fig. 1.**
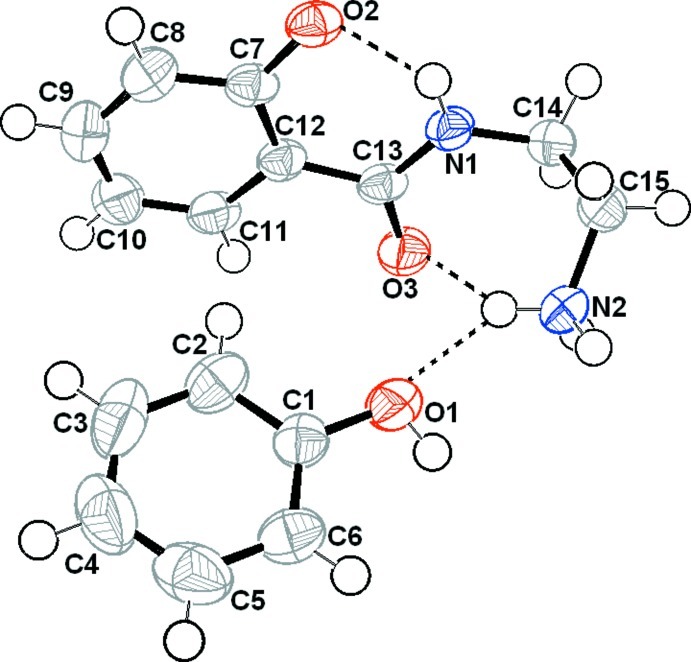
The asymmetric unit of (I) with displacement ellipsoids drawn at the 50% probability level.

**Fig. 2.**
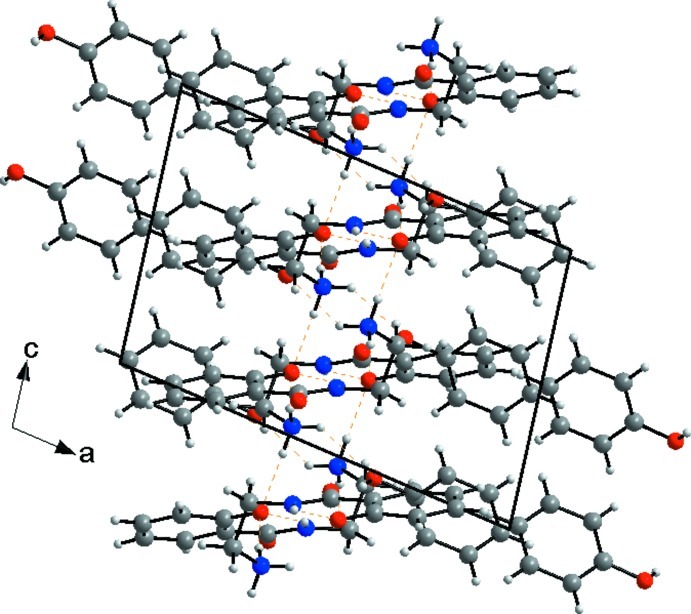
Alternating layers of (I) viewed via b axis showing hydrogen bonds as dashed lines [O—H···O and N—H···O interactions].

## supplementary crystallographic information

### Comment

Salicylic amide with its diverse amidic forms namely ethylelenediamine or other amines were found to be as good chelating agents currently applied in coordination chemistry (Koch, 2001; Hancock *et al.*, 1989).

The molecule structure of (I), is illustrated in Fig. 1. In the title structure the phenol molecule is cocrystalized with ethylenediamine Salicylic amide The crystal packing can be described by layers parallel to (100) planes (Fig. 2). It features intermolecular O—H···O and N—H···O hydrogen bonds (Fig. 2, Table 1). These interactions link the molecules within the layers and also link the layers together.

### Experimental

0.06 g (1 mmol) ethylenediamine was dissolved in 20 ml of methanol. To this methanolic solution 0.214 g (1 mmol) of phenyl salicylate were added in one portion. This mixture was stirred for one hour at room temperature, and then 0.172 g (1 mmol) of 2-hydroxynaphtaldehyde were also added and heated to 60 °C for 4 h. The solid obtained was recovered by filtration after reducing of its volume on vaccum with rotating evaporator to obtain colourless prisms.

### Refinement

The H atoms were localized on Fourier maps but introduced in calculated positions and treated as riding on their parent atom (C,*O* and N) with C—H = 0.97 Å (ethylene)or 0.93 Å (aromatic), O—H = 0.82 Å and N—H = 0.86 Å or 0.89 Å (ammonium); with *U*_iso_(H) = 1.2*U*_eq_(ammonium and hydroxy) and *U*_iso_(H) = 1.5*U*_eq_.

### Crystal data

**Table:**

C_9_H_12_N_2_O_2_·C_6_H_6_O	*F*(000) = 584
*M**_r_* = 274.31	*D*_x_ = 1.298 Mg m^−^^3^
Monoclinic, *P*2_1_/*c*	Mo *K*α radiation, λ = 0.71073 Å
Hall symbol: -P 2ybc	Cell parameters from 4306 reflections
*a* = 12.6494 (4) Å	θ = 2.9–27.4°
*b* = 13.2145 (6) Å	µ = 0.09 mm^−^^1^
*c* = 8.5445 (4) Å	*T* = 150 K
β = 100.637 (2)°	Prism, colorless
*V* = 1403.72 (10) Å^3^	0.58 × 0.52 × 0.38 mm
*Z* = 4	

### Data collection

**Table:**

Bruker APEXII CCD diffractometer	2649 reflections with *I* > 2σ(*I*)
Graphite monochromator	*R*_int_ = 0.035
CCD rotation images, thin slices scans	θ_max_ = 27.5°, θ_min_ = 2.9°
Absorption correction: multi-scan (*SADABS*; Bruker, 2011)	*h* = −16→16
*T*_min_ = 0.860, *T*_max_ = 0.966	*k* = −12→17
12244 measured reflections	*l* = −11→11
3208 independent reflections	

### Refinement

**Table:**

Refinement on *F*^2^	Primary atom site location: structure-invariant direct methods
Least-squares matrix: full	Secondary atom site location: difference Fourier map
*R*[*F*^2^ > 2σ(*F*^2^)] = 0.040	Hydrogen site location: inferred from neighbouring sites
*wR*(*F*^2^) = 0.102	H-atom parameters constrained
*S* = 1.03	*w* = 1/[σ^2^(*F*_o_^2^) + (0.0448*P*)^2^ + 0.4498*P*] where *P* = (*F*_o_^2^ + 2*F*_c_^2^)/3
3208 reflections	(Δ/σ)_max_ < 0.001
181 parameters	Δρ_max_ = 0.27 e Å^−^^3^
0 restraints	Δρ_min_ = −0.21 e Å^−^^3^

### Special details

**Table:**

Geometry. All e.s.d.'s (except the e.s.d. in the dihedral angle between two l.s. planes) are estimated using the full covariance matrix. The cell e.s.d.'s are taken into account individually in the estimation of e.s.d.'s in distances, angles and torsion angles; correlations between e.s.d.'s in cell parameters are only used when they are defined by crystal symmetry. An approximate (isotropic) treatment of cell e.s.d.'s is used for estimating e.s.d.'s involving l.s. planes.
Refinement. Refinement of *F*^2^ against ALL reflections. The weighted *R*-factor *wR* and goodness of fit *S* are based on *F*^2^, conventional *R*-factors *R* are based on *F*, with *F* set to zero for negative *F*^2^. The threshold expression of *F*^2^ > σ(*F*^2^) is used only for calculating *R*-factors(gt) *etc*. and is not relevant to the choice of reflections for refinement. *R*-factors based on *F*^2^ are statistically about twice as large as those based on *F*, and *R*- factors based on ALL data will be even larger.

### Fractional atomic coordinates and isotropic or equivalent isotropic displacement parameters (Å^2^)

**Table:**

	*x*	*y*	*z*	*U*_iso_*/*U*_eq_	
O2	0.59307 (7)	0.65808 (6)	0.29145 (10)	0.0207 (2)	
O3	0.56158 (7)	0.34524 (7)	0.37386 (11)	0.0260 (2)	
N1	0.47865 (8)	0.48940 (8)	0.27880 (12)	0.0195 (2)	
H1N	0.4845	0.5532	0.2627	0.023*	
N2	0.43633 (8)	0.32203 (8)	0.03416 (11)	0.0186 (2)	
H2A	0.5036	0.3421	0.0701	0.022*	
H2B	0.4287	0.306	−0.0684	0.022*	
H2C	0.4219	0.2681	0.0891	0.022*	
C7	0.67497 (10)	0.60303 (9)	0.36441 (13)	0.0192 (3)	
C8	0.77747 (11)	0.64776 (10)	0.40959 (17)	0.0290 (3)	
H8	0.7869	0.7148	0.3819	0.035*	
C9	0.86393 (11)	0.59533 (11)	0.49331 (19)	0.0338 (3)	
H9	0.9302	0.6274	0.5215	0.041*	
C10	0.85282 (11)	0.49477 (11)	0.53593 (17)	0.0302 (3)	
H10	0.9106	0.4599	0.595	0.036*	
C11	0.75500 (10)	0.44784 (10)	0.48917 (14)	0.0227 (3)	
H11	0.7478	0.3803	0.5162	0.027*	
C12	0.66564 (10)	0.49853 (9)	0.40198 (13)	0.0183 (3)	
C13	0.56553 (10)	0.43867 (9)	0.35241 (13)	0.0186 (3)	
C14	0.37497 (10)	0.44126 (9)	0.22499 (14)	0.0201 (3)	
H14A	0.3182	0.4889	0.2346	0.024*	
H14B	0.3681	0.3839	0.2933	0.024*	
C15	0.36081 (10)	0.40533 (9)	0.05392 (14)	0.0202 (3)	
H15A	0.2875	0.382	0.0195	0.024*	
H15B	0.3726	0.4617	−0.0135	0.024*	
O1	0.65559 (7)	0.32899 (7)	−0.02109 (12)	0.0300 (2)	
H1	0.6334	0.2758	−0.0644	0.045*	
C1	0.76300 (10)	0.33979 (9)	−0.02608 (15)	0.0212 (3)	
C2	0.82529 (11)	0.39958 (10)	0.08869 (16)	0.0272 (3)	
H2	0.7942	0.4332	0.1645	0.033*	
C3	0.93442 (12)	0.40858 (11)	0.08896 (19)	0.0368 (4)	
H3	0.9769	0.4482	0.1659	0.044*	
C4	0.98085 (12)	0.35932 (12)	−0.0238 (2)	0.0418 (4)	
H4	1.0544	0.3648	−0.0217	0.05*	
C5	0.91760 (12)	0.30184 (11)	−0.1397 (2)	0.0374 (4)	
H5	0.9485	0.2695	−0.2168	0.045*	
C6	0.80857 (11)	0.29210 (10)	−0.14187 (16)	0.0265 (3)	
H6	0.766	0.2538	−0.2206	0.032*	

### Atomic displacement parameters (Å^2^)

**Table:**

	*U*^11^	*U*^22^	*U*^33^	*U*^12^	*U*^13^	*U*^23^
O2	0.0266 (5)	0.0161 (4)	0.0188 (4)	0.0027 (3)	0.0023 (3)	0.0004 (3)
O3	0.0273 (5)	0.0163 (4)	0.0321 (5)	0.0016 (4)	−0.0003 (4)	0.0016 (4)
N1	0.0217 (5)	0.0143 (5)	0.0213 (5)	0.0006 (4)	0.0008 (4)	−0.0005 (4)
N2	0.0217 (5)	0.0175 (5)	0.0163 (5)	−0.0020 (4)	0.0026 (4)	−0.0013 (4)
C7	0.0242 (6)	0.0188 (6)	0.0148 (5)	0.0032 (5)	0.0042 (4)	−0.0022 (4)
C8	0.0284 (7)	0.0205 (7)	0.0378 (8)	−0.0028 (5)	0.0052 (6)	−0.0014 (5)
C9	0.0219 (7)	0.0310 (8)	0.0462 (9)	−0.0034 (6)	−0.0001 (6)	−0.0067 (6)
C10	0.0250 (7)	0.0284 (7)	0.0332 (7)	0.0060 (6)	−0.0050 (5)	−0.0052 (6)
C11	0.0274 (7)	0.0189 (6)	0.0203 (6)	0.0038 (5)	0.0009 (5)	−0.0030 (5)
C12	0.0222 (6)	0.0177 (6)	0.0150 (5)	0.0014 (5)	0.0030 (4)	−0.0028 (4)
C13	0.0240 (6)	0.0168 (6)	0.0147 (5)	0.0023 (5)	0.0029 (4)	−0.0021 (4)
C14	0.0201 (6)	0.0192 (6)	0.0209 (6)	0.0014 (5)	0.0033 (5)	−0.0013 (5)
C15	0.0211 (6)	0.0177 (6)	0.0203 (6)	0.0018 (5)	0.0004 (4)	0.0015 (5)
O1	0.0244 (5)	0.0233 (5)	0.0436 (6)	−0.0046 (4)	0.0096 (4)	−0.0114 (4)
C1	0.0237 (6)	0.0157 (6)	0.0240 (6)	−0.0004 (5)	0.0033 (5)	0.0034 (5)
C2	0.0358 (8)	0.0214 (6)	0.0235 (6)	−0.0041 (6)	0.0030 (5)	0.0011 (5)
C3	0.0340 (8)	0.0288 (8)	0.0420 (8)	−0.0099 (6)	−0.0076 (6)	0.0050 (6)
C4	0.0240 (7)	0.0311 (8)	0.0709 (11)	−0.0010 (6)	0.0101 (7)	0.0105 (8)
C5	0.0388 (8)	0.0247 (7)	0.0543 (10)	0.0025 (6)	0.0232 (7)	0.0029 (7)
C6	0.0338 (7)	0.0192 (6)	0.0272 (7)	−0.0009 (5)	0.0074 (5)	−0.0004 (5)

### Geometric parameters (Å, º)

**Table:**

O2—C7	1.3239 (15)	C12—C13	1.4868 (17)
O3—C13	1.2505 (15)	C14—C15	1.5158 (16)
N1—C13	1.3408 (16)	C14—H14A	0.97
N1—C14	1.4537 (15)	C14—H14B	0.97
N1—H1N	0.86	C15—H15A	0.97
N2—C15	1.4874 (16)	C15—H15B	0.97
N2—H2A	0.8899	O1—C1	1.3747 (15)
N2—H2B	0.8897	O1—H1	0.8195
N2—H2C	0.8904	C1—C6	1.3852 (18)
C7—C8	1.4123 (18)	C1—C2	1.3862 (18)
C7—C12	1.4276 (17)	C2—C3	1.385 (2)
C8—C9	1.378 (2)	C2—H2	0.93
C8—H8	0.93	C3—C4	1.381 (2)
C9—C10	1.392 (2)	C3—H3	0.93
C9—H9	0.93	C4—C5	1.380 (2)
C10—C11	1.3758 (19)	C4—H4	0.93
C10—H10	0.93	C5—C6	1.382 (2)
C11—C12	1.4037 (17)	C5—H5	0.93
C11—H11	0.93	C6—H6	0.93
			
C13—N1—C14	122.89 (10)	N1—C14—H14A	109.1
C13—N1—H1N	118.6	C15—C14—H14A	109.1
C14—N1—H1N	118.5	N1—C14—H14B	109.1
C15—N2—H2A	109.5	C15—C14—H14B	109.1
C15—N2—H2B	109.4	H14A—C14—H14B	107.9
H2A—N2—H2B	109.5	N2—C15—C14	112.16 (10)
C15—N2—H2C	109.5	N2—C15—H15A	109.2
H2A—N2—H2C	109.5	C14—C15—H15A	109.2
H2B—N2—H2C	109.4	N2—C15—H15B	109.2
O2—C7—C8	119.86 (11)	C14—C15—H15B	109.2
O2—C7—C12	123.18 (11)	H15A—C15—H15B	107.9
C8—C7—C12	116.95 (11)	C1—O1—H1	109.5
C9—C8—C7	122.14 (13)	O1—C1—C6	121.26 (12)
C9—C8—H8	118.9	O1—C1—C2	118.24 (12)
C7—C8—H8	118.9	C6—C1—C2	120.50 (12)
C8—C9—C10	120.46 (13)	C3—C2—C1	119.09 (13)
C8—C9—H9	119.8	C3—C2—H2	120.5
C10—C9—H9	119.8	C1—C2—H2	120.5
C11—C10—C9	118.90 (13)	C4—C3—C2	120.70 (14)
C11—C10—H10	120.5	C4—C3—H3	119.6
C9—C10—H10	120.5	C2—C3—H3	119.6
C10—C11—C12	122.14 (12)	C5—C4—C3	119.69 (14)
C10—C11—H11	118.9	C5—C4—H4	120.2
C12—C11—H11	118.9	C3—C4—H4	120.2
C11—C12—C7	119.27 (11)	C4—C5—C6	120.38 (14)
C11—C12—C13	117.22 (11)	C4—C5—H5	119.8
C7—C12—C13	123.50 (11)	C6—C5—H5	119.8
O3—C13—N1	120.72 (11)	C5—C6—C1	119.60 (13)
O3—C13—C12	122.58 (11)	C5—C6—H6	120.2
N1—C13—C12	116.68 (11)	C1—C6—H6	120.2
N1—C14—C15	112.41 (10)		
			
O2—C7—C8—C9	176.44 (12)	C7—C12—C13—O3	173.39 (11)
C12—C7—C8—C9	−3.24 (19)	C11—C12—C13—N1	175.56 (10)
C7—C8—C9—C10	0.3 (2)	C7—C12—C13—N1	−4.97 (16)
C8—C9—C10—C11	1.8 (2)	C13—N1—C14—C15	−92.17 (13)
C9—C10—C11—C12	−1.0 (2)	N1—C14—C15—N2	66.25 (13)
C10—C11—C12—C7	−2.00 (18)	O1—C1—C2—C3	177.79 (12)
C10—C11—C12—C13	177.50 (11)	C6—C1—C2—C3	−1.91 (19)
O2—C7—C12—C11	−175.68 (11)	C1—C2—C3—C4	0.4 (2)
C8—C7—C12—C11	3.98 (16)	C2—C3—C4—C5	1.1 (2)
O2—C7—C12—C13	4.86 (17)	C3—C4—C5—C6	−1.0 (2)
C8—C7—C12—C13	−175.47 (11)	C4—C5—C6—C1	−0.5 (2)
C14—N1—C13—O3	2.23 (17)	O1—C1—C6—C5	−177.71 (12)
C14—N1—C13—C12	−179.37 (10)	C2—C1—C6—C5	1.98 (19)
C11—C12—C13—O3	−6.08 (17)		

### Hydrogen-bond geometry (Å, º)

**Table:**

*D*—H···*A*	*D*—H	H···*A*	*D*···*A*	*D*—H···*A*
O1—H1···O3^i^	0.82	1.87	2.6696 (13)	166
N1—H1*N*···O2	0.86	1.93	2.6490 (13)	140
N2—H2*A*···O1	0.89	2.21	2.8995 (14)	134
N2—H2*A*···O3	0.89	2.56	3.0547 (13)	116
N2—H2*B*···O2^ii^	0.89	1.93	2.7506 (13)	152
N2—H2*C*···O2^iii^	0.89	1.81	2.6939 (13)	174

Symmetry codes: (i) *x*, −*y*+1/2, *z*−1/2; (ii) −*x*+1, −*y*+1, −*z*; (iii) −*x*+1, *y*−1/2, −*z*+1/2.

## References

[bb1] Brandenburg, K. & Berndt, M. (2001). *DIAMOND* Crystal Impact, Bonn, Germany.

[bb2] Bruker (2011). *APEX2*, *SAINT* and *SADABS* Bruker AXS Inc., Madison, Wisconsin, USA.

[bb3] Burla, M. C., Caliandro, R., Camalli, M., Carrozzini, B., Cascarano, G. L., De Caro, L., Giacovazzo, C., Polidori, G. & Spagna, R. (2005). *J. Appl. Cryst.* **38**, 381–388.

[bb4] Farrugia, L. J. (2012). *J. Appl. Cryst.* **45**, 849–854.

[bb5] Hancock, R. D. & Martell, A. E. (1989). *Chem. Rev.* **89**, 1875–1914.

[bb6] Koch, K. R. (2001). *Coord. Chem. Rev.* **216**, 473–488.

[bb7] Sheldrick, G. M. (2008). *Acta Cryst.* A**64**, 112–122. 10.1107/S0108767307043930 18156677

